# A Manual of Surgical Treatment

**Published:** 1901-07

**Authors:** 


### Book Reviews.

A MANUAL OF SURGICAL TREATMENT. By W. WATSON CHEYNE, M. B., F. R. C. S., F. R. S , Professor of Surgery in King's College, London; Surgeon to King's College Hospital, etc; and F. F. BURGHARD, M. D. and M. S (Lond.), F. R. C. S , Teacher of Practical Surgery in King's College, London; Surgeon to King's College Hospital, etc. In seven imperial 8vo volumes, with illustrations. Volume IV , 383 pages with 138 illustrations. Philadelphia and New York: Lea Brothers & Co. 1900. [Cloth, $3.75 net.]

The fourth volume of this very excellent work on surgery bears out the promises made by the authors in the preface to the first book, and as the work goes on through successive volumes, it becomes more and more apparent that an ideal work on surgery is being given us. It is one which, as has been stated before, is comprehensive and practical. It deals not with the ginger-bread theories of surgery nor does it advise doing this, that, or the other because some one else has done it, or because it's pretty or theatric. On the contrary everything written of and every practice recommended has the stamp of common sense and the proof of long practice—and what is better than all, excellent result. Cheyne and Burghard are men of careful procedure and wide experience. They know what they do in surgery and why they do it, and they make it exceedingly clear to the reader that the best result in a given case can be obtained by following a certain procedure, which is laid down concisely and clearly without any frills or rhetoric.

Volume IV. deals with the treatment of the surgical affections of the joints—including excisions, and the spine—most difficult subjects to intelligently place before general readers How true this is, may be gathered from even a cursory skimming over of some of the standard works on surgery. Yet the same agreeable simplicity and modesty marks the handling of this volume as was so apparent—so refreshing—in the previous books by these authors. This trait is so strongly marked in no volume thus far issued as in the present, where they publicly acknowledge the reproduction in full of Dr. Percy Lewis's splendid manual of medical exercises, which is used as an appendix to the chapter on scoliosis. This is taken from Lewis's book, On the Relief and Cure of Spinal Curvature. So excellent is this work deemed by Cheyne and Burghard that they have reproduced the illustrations.

Another feature of this volume is the contribution of Dr. Leslie Walsh, of Bath, who has furnished a most practical account of the thermal treatment of rheumatoid arthritis, as practised at Bath. This is not only helpful but is extremely interesting.

There is in the preface a note of regret on the part of the writers having been reproached by "several critics" for not mentioning "certain methods of treatment to which they are personally addicted." Such criticism at best is narrow-minded, for it was explicitly stated in the first volume that the work would be largely confined to procedures which had proved most efficient in their hands. Some of the more recent methods of treatment may in time prove of great value, but until they are proven by accurate results to be better than the treatment laid down by Cheyne and Burghard, the latter cannot be consistently blamed for not using or recommending or even mentioning them. If they once started in to hand out literary bouquets to all the faddists in the field of surgery, they would be writing a very cumbersome and homely and useless encyclopedia, which, while a curiosity, would be nothing more or less than surgical rag-bag. Instead, they are getting out a very handsome and useful manual in seven volumes. Under the division devoted to surgical affection of the joints are considered injuries, such as dislocations, each of which is taken up in detail; sprains of joints and the wounds of joints. Under diseases of joints are the inflammations, tuberculosis, syphilis, nervous affections, torn bodies and rheumatoid arthritis.

The diseases of individual joints is most comprehensive and a great deal of attention is paid to careful diagnosis. The surgical affection of the spine includes injuries of all characters, spina bifida, kyphosis, scoliosis, tuberculosis, spondylitis deformans, acute osteomyelitis, actinomycosis, new growths, hysterical spine and sacro-coccygeal tumors.

The same uniformity of thought which has done much to place the whole work in the front rank of medical literature, characterises this book, and one is naturally lead to look forward to the forthcoming volumes with pleasurable anticipation. N. W. W.

